# Neuroprotective Effect of Artesunate in Experimental Model of Traumatic Brain Injury

**DOI:** 10.3389/fneur.2018.00590

**Published:** 2018-07-31

**Authors:** Enrico Gugliandolo, Ramona D'Amico, Marika Cordaro, Roberta Fusco, Rosalba Siracusa, Rosalia Crupi, Daniela Impellizzeri, Salvatore Cuzzocrea, Rosanna Di Paola

**Affiliations:** ^1^Department of Chemical, Biological, Pharmaceutical and Environmental Sciences, University of Messina, Messina, Italy; ^2^Department of Pharmacological and Physiological Science, Saint Louis University, St. Louis, MO, United States

**Keywords:** traumatic brain injury, neuroinflammation, neurodegeneration, artesunate, artemisin

## Abstract

Traumatic brain injuries (TBI) are an important public health challenge. In addition, subsequent events at TBI can compromise the quality of life of these patients. In fact, TBI is associated with several complications for both long and short term, some evidence shows how TBI is associated with a decline in cognitive functions such as the risk of developing dementia, cerebral atrophy, and Parkinson disease. After the direct damage from TBI, a key role in TBI injury is played by the inflammatory response and oxidative stress, that contributes to tissue damage and to neurodegenerative processes, typical of secondary injury, after TBI. Given the complex series of events that are involved after TBI injury, a multitarget pharmacological approach is needed. Artesunate is a more stable derivative of its precursor artemisin, a sesquiterpene lactone obtained from a Chinese plant *Artemisia annua*, a plant used for centuries in traditional Chinese medicine. artesunate has been shown to be a pluripotent agent with different pharmacological actions. therefore, in this experimental model of TBI we evaluated whether the treatment with artesunate at the dose of 30 mg\Kg, had an efficacy in reducing the neuroinflammatory process after TBI injury, and in inhibiting the NLRP3 inflammasome pathway, which plays a key role in the inflammatory process. We also assessed whether treatment with artesunate was able to exert a neuroprotective action by modulating the release of neurotrophic factors. our results show that artesunate was able to reduce the TBI-induced lesion, it also showed an anti-inflammatory action through the inhibition of Nf-kb, release of proinflammatory cytokines IL-1β and TNF-α and through the inhibition NLRP3 inflammasome complex, furthermore was able to reduce the activation of astrocytes and microglia (GFAP, Iba-1). Finally, our results show that the protective effects of artesunate also occur through the modulation of neurotrophic factors (BDNF, GDNF, NT-3) that play a key role in neuronal survival.

## Introduction

Artemisinin is a sesquiterpene lactone obtained from a Chinese plant *Artemisia annua* a plant used for centuries in traditional Chinese medicine (1) and known for its antimalarial properties (2). Artesunate is a more stable derivative of its precursor artemisin, and is considered more the most effective drug for treating severe and chloroquine-resistant malaria, seen also its excellent safety profile (3, 4). Artesunate also has anti-tumor properties and thanks to its good tolerability profile is used in combination with standard chemotherapeutic (5, 6). Therefore, artesunate has been shown to be a pluripotent agent with different pharmacological actions, in fact in addition to these properties, has also shown to have an anti-inflammatory activity, in different inflammatory model like allergic asthma (7) and sepsis (8). Artesunate is the most effective drug for the treatment of cerebral malaria, in addition recent studies show that the treatment with artesunate also reduces inflammation of the brain, associated with this disease(9, 10). Artesunate is able to reach and maintain a high concentration in the brain, and this makes it a good candidate for the treatment of diseases of the central nervous system and neurological disorders (11), as an anti-neuroinflammatory agent (12). Traumatic brain injuries (TBI) is a major public health challenge and may affect people in a wide range of ages. In addition, events following TBI can compromise the quality of life of these patients (13). In fact, TBI is associated with several complications both long and short term (14). In fact, some evidence shows how TBI is associated with a decline in cognitive functions (15, 16), such as the risk of developing dementia and cerebral atrophy (17) and can increase the risk to developed Parkinson's disease (PD) (18). The damage from TBI is partly due to a direct consequence of the primary injury, and later with an indirect mechanism (secondary injury). Indeed, different mechanisms contribute to secondary damage. A key role is played by the acute inflammatory response, with the release of many inflammation mediators and the activation of different pro-inflammatory pathways, such as NLRP3 inflammasome (19). Another significant contribution to secondary damage is given by oxidative stress (20). For this series of complex events that are involved in TBI injury, a multi-target pharmacological treatment is necessary. Therefore, as artesunate is compound with more than one protective effect, the aim of this paper was to study if artesunate should be a powerful candidate for the treatment of brain trauma.

## Materials and methods

### Animals

Male CD1 mice (25-30 g, Envigo, Italy), aged between 10 and 12 weeks, were used for all studies. Mice were placed in a controlled location with standard rodent chow and water. Animals were kept at 22 ± 1°C with a 12-h light, 12-h dark cycle. The study was permitted by the University of Messina Review Board for the care of animals. All animal experiments were performed following the regulations in Italy (D.M. 116192), Europe (O.J. of E.C. L 358/1 12/18/1986).

### Controlled cortical impact (CCI) experimental traumatic brain injury TBI

Mice were anesthetized under intraperitoneal (i.p.) ketamine and xylazine (2.6 and 0.16 mg/kg body weight, respectively). TBI was induced in mice by a controlled cortical impactor (CCI) as previously described (21) In brief, a craniotomy was made in the right hemisphere, encompassing bregma and lambda, and between the sagittal suture and the coronal ridge, with a Micro motor hand piece and drill. The resulting bone flap was removed, and the craniotomy enlarged further. A cortical contusion was produced on the exposed cortex using the controlled impactor device Impact OneTM Stereotaxic impactor for CCI (Leica, Milan, Italy), the impact tip was centered and lowered over the craniotomy site until it touched the dura mater. Then, the rod was retracted, and the impact tip was advanced farther to produce a brain injury of moderate severity for mice (tip diameter: 4 mm; cortical contusion depth: 3 mm; impact velocity: 1.5 m/s). Immediately after injury, the skin incision was closed with nylon sutures, and 2% lidocaine jelly was applied to the lesion site to minimize any possible discomfort.

### Experimental groups

Mice were randomly distributed into the following groups: (*n* = 10 for each group, calculated using the statistical test a priori power analyzes of the G-power software)

TBI + vehicle: mice (*n* = 10) were subjected to CCI and vehicle (saline) was administered at 1 after craniotomy.
TBI + Artesunate: mice (*n* = 10) were subjected to CCI and Artesunate (30 mg/kg) was administered at 1 after craniotomy.
Sham + vehicle: mice (*n* = 10) were subjected to the surgical procedures as above group (anesthesia and craniotomy) except that the impact tip was not applied, and vehicle was administered at 1 after craniotomy.
Sham + artesunate: mice (*n* = 10) were subjected to the surgical procedures as above group (anesthesia and craniotomy) except that the impact tip was not applied and Artesunate (30 mg/kg) was administered at 1 after craniotomy.
As describe below mice (*n* = 10 from each group for each parameters) were sacrificed at 24 h after TBI to evaluate the various parameter.

### Histology

Coronal sections of 5-μm thickness were sectioned from the perilesional brain area of each animal and were evaluated by an experienced histopathologist. Damaged neurons were counted and the histopathologic changes of the gray matter were scored on a six-point scale (22): 0, no lesion observed; 1, gray matter contained one to five eosinophilic neurons; 2, gray matter contained five to 10 eosinophilic neurons; 3, gray matter contained more than 10 eosinophilic neurons; 4, small infarction (less than one third of the gray matter area); 5, moderate infarction (one third to one half of the gray matter area); 6, large infarction (more than half of the gray matter area). The scores from all the sections of each brain were averaged to give a final score for individual mice. All the histological studies were performed in a blinded fashion.

### Assessment of lesion volume

At 24 h after injuries, mice were euthanized, and brains were frozen. The brains were sectioned in coronal sections (14 μm). Sections were stained with hematoxylin. Area of the undamaged and injured hemisphere was measured on each section using image analysis software. The hemispheric volume was obtained by summing area of each section and multiplying it by 0.5. Lesion volume (mm3) was expressed as difference between the uninjured and injured hemisphere volume.

### Western blot analysis

Western blot analysis was performed on tissues harvested 24 h post-TBI. Cytosolic and nuclear extracts were prepared as described previously (23). The filters were probed with specific Abs: anti- NF-κB p-65 (1:1,000; Santa Cruz Biotechnology) or IκB-α (1:1,000; Santa Cruz Biotechnology), or anti-NLRP3(1:500; Santa Cruz Biotechnology), or anti BAX (1:500; Santa Cruz Biotechnology) anti-Bcl-2 (1:500; Santa Cruz Biotechnology) anti-ASC (1:500; Santa Cruz Biotechnology), or anti Caspase-1 (1:500; Santa Cruz Biotechnology) in 1 × PBS, 5% w/v nonfat dried milk, 0.1% Tween-20 at 4°C, overnight. To ascertain that blots were loaded with equal amounts of proteins they were also incubated in the presence of the antibody against β-actin protein (cytosolic fraction 1:500; Santa Cruz Biotechnology) or lamin A/C (nuclear fraction 1:500 Sigma–Aldrich Corp.). Signals were detected with enhanced chemiluminescence (ECL) detection system reagent according to the manufacturer's instructions (Thermo, USA). The relative expression of the protein bands was quantified by densitometry with BIORAD ChemiDocTM XRS+software and standardized to β-actin and lamin A/C levels. Images of blot signals (8 bit/600 dpi resolution) were imported to analysis software (Image Quant TL,v2003). The blot was stripped with glycine 2% and reproved several times to optimize detection of proteins and to visualize other proteins without the need for multiple gels and transfers.

### Immunohistochemistry

Tissue segments containing the lesion (1 cm on each side of the lesion) were fixed in 10% (w/v) buffered formaldehyde 24 h after TBI and sliced in 7-μm sections for paraffin- embedding previously described (24). After deparaffinization, endogenous peroxidase was quenched with 0.30% (v/v) hydrogen peroxide in 60% (v/v) methanol for 30 min. The sections were permeabilized with 0.1% (w/v) Triton X-100 in PBS for 20 min. Non-specific adsorption was minimized by incubating the section in 2% (v/v) normal goat serum in PBS for 20 min. Endogenous biotin or avidin binding sites were blocked by sequential incubation for 15 min with biotin and avidin, respectively. Afterwards, the sections were incubated overnight with one of the following primary antibodies diluted in PBS: polyclonal anti-glial cell line-derived neurotrophic factor (GDNF) (1:500, Santa Cruz Biotechnology), polyclonal anti-brain-derived neurotrophic factor (BDNF) (1:500, Santa Cruz Biotechnology), anti- IL-1β (1:500, Santa Cruz Biotechnology), anti-TNF-α (1:500, Santa Cruz Biotechnology), anti-iNOS. (1:500, Santa Cruz Biotechnology), anti-VEGF (1:500, Santa Cruz Biotechnology). The immunohistochemical images were collected by Zeiss microscope using Axio Vision software. For graphic representation of densitometric analyses, we measured the intensity of positive staining (brown staining) by computer-assisted color image analysis (Leica QWin V3, UK). The percentage area of immunoreactivity (determined by the number of positive pixels) was expressed as percent of total tissue area (red staining) as seen previously (18).

### Immunofluorescence

After deparaffinization and rehydration, detection of GFAP, Iba1, neurotrophin-3 (NT-3), BDNF, GDNF, IL-1β was carried out after boiling the tissue sections in 0.1 M citrate buffer for 1 min as described previously (18). Non-specific adsorption was minimized by incubating in 2% (vol/vol) normal goat serum in PBS for 20 min. Sections were incubated with one of the following primary antibodies: rabbit polyclonal anti- GFAP (1:100, Santa Cruz Biotechnology), rabbit polyclonal anti- Iba1 (1:100, Santa Cruz Biotechnology), rabbit polyclonal anti-NT3 (1:100, Millipore), polyclonal anti-glial cell line-derived neurotrophic factor (GDNF) (1:100, Santa Cruz Biotechnology), polyclonal anti-brain-derived neurotrophic factor (BDNF) (1:100, Santa Cruz Biotechnology), anti- IL-1β (1:100, Santa Cruz Biotechnology) in a humidified oxygen and nitrogen chamber overnight at 37°C. Sections were then incubated with secondary antibody: fluorescein isothiocyanate-conjugated anti- mouse Alexa Fluor-488 (1:2,000, Molecular Probes, Monza, Italy) or Texas Red-conjugated anti-rabbit Alexa Fluor-594 (1:1,000, Molecular Probes) for 1 h at 37°C. For nuclear staining, 2 μg/ml 4′, 6′ -diamidino-2-phenylindole (DAPI; Hoechst, Frankfurt, Germany) in PBS was added. Sections were observed using a Leica DM2000 microscope (Leica, Milan, Italy). GFAP+, Iba1+, and NT- 3+, BDNF+, GDNF+, IL-1β+ cells were counted stereologically on sections cut at a 40 μm thickness and every 4th section was counted using a grid of 100 × 100 μm. Optical sections of fluorescence specimens were obtained using a HeNe laser (543 nm), an ultraviolet laser (361–365 nm) and an argon laser (458 nm) at a one-mi, 2s scanning speed with up to eight averages; 1.5 μm sections were obtained using a pinhole of 250. The same settings were used for all images obtained from the other samples that had been processed in parallel. Digital images were cropped, and figure montages prepared using Adobe Photoshop 7.0 (Adobe Systems; Palo Alto, California, United States). Both brightfield (NeuN or BDNF or GDNF or NT3 or GFAP or IBA1 or IL1β) and fluorescent photographs (DAPI) were taken of 3 representative fields per slide in a blinded fashion using a fluorescent microscope (Leica DM 2000, 100 × objective). The total number of nuclei per field were quantified by counting the DAPI-positive nuclei using ImageJ software (25–29).

### Materials

Artesunate was obtained by Santa Cruz Biotechnology. All compounds used in this study, except where differently specified, were purchased from Sigma-Aldrich Company Ltd. All solutions used for *in vivo* administrations were made using no pyrogenic saline (0.9% wt/vol NaCl; Baxter Healthcare Ltd., Thetford, Norfolk, UK).

### Statistical analysis

All values in the figures and text are expressed as mean ± standard error of the mean (SEM) of N number of animals. In those experiments involving histology or immunohistochemistry, the pictures exhibited are representative of at least three experiments performed on different days. Results were analyzed by one-way ANOVA followed by a Bonferroni *post-hoc* test for multiple comparisons. Histological score was analyzed by Kruskal-Wallis test followed by a Dunn's multiple comparisons test. A *p*-value < 0.05 was considered significant. For one-way ANOVA statistic test, a single “F” value indicated as variation between sample means/variation within the samples was shown.

## Results

### Protective effect of artesunate treatment on histological analysis after TBI

**Figure 1:**
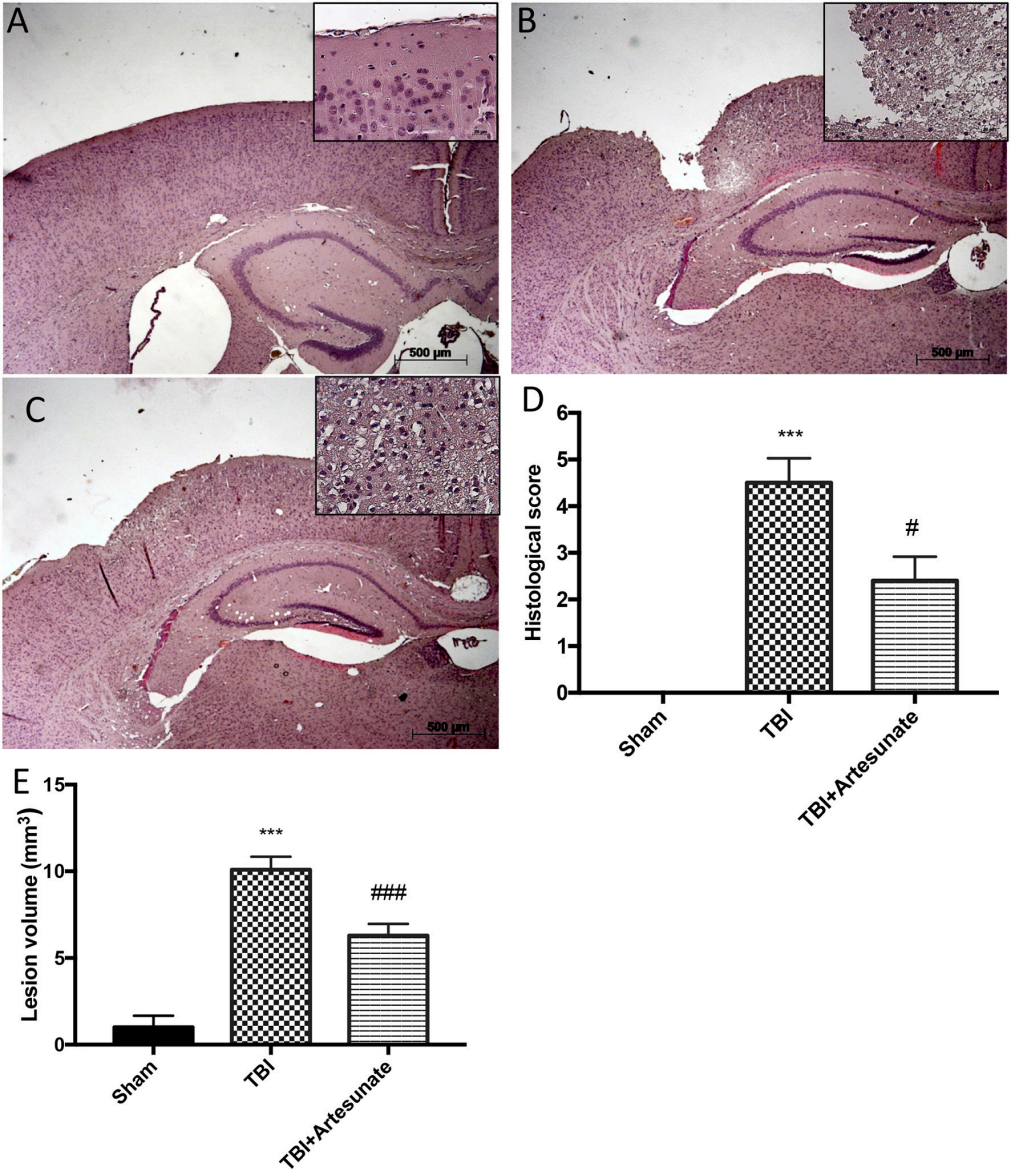
Protective effect of artesunate treatment on histological analysis after TBI. **(A)** Show brain section from mice of sham group. Brain section at the level of the perilesional area showed that 24 h after TBI injury the TBI group showed an evident tissue disorganization and inflammation in the perilesional area **(B)**. The group that has been treated with artesunate **(C)** at dose of 30 mg\Kg showed a significant reduction in degree of brain injury compared with the TBI group **(D)**. Moreover treatment with artesunate significant reduce the lesion volume compared to TBI group as show in **(E)**. Data are expressed as Mean ± SEM from *N* = 10 Mice for each group, ***P < 0.001 vs. corresponding sham group. ^#^*P* < 0.05 vs. corresponding TBI. ^###^*P* < 0.001 vs. corresponding TBI. (*F*-value for histological score = 279.4. *df* = 2. *r*^2^ = 0.9539) (*F*-value for lesion volume = 433.9. *df* = 2. *r*^2^ = 0.9698).

Histological analysis of brain section at the level of the perilesional area showed that 24 h after TBI injury, in the TBI group there is a significant tissue damage, inflammation in, compared with brain from sham group (Figure 1A) the perilesional area and white matter alteration (Figure 1B). The treatments with artesunate (30 mg\Kg) significantly reduce the degree of brain injury when compared to the TBI group, (Figure 1C see histological score Figure 1D). Moreover, treatment with artesunate significantly reduce the lesion volume compared to the TBI group (Figure 1E).

### Protective effect of artesunate treatment on Iκb-α degradation, Nf-κb translocation

**Figure 2:**
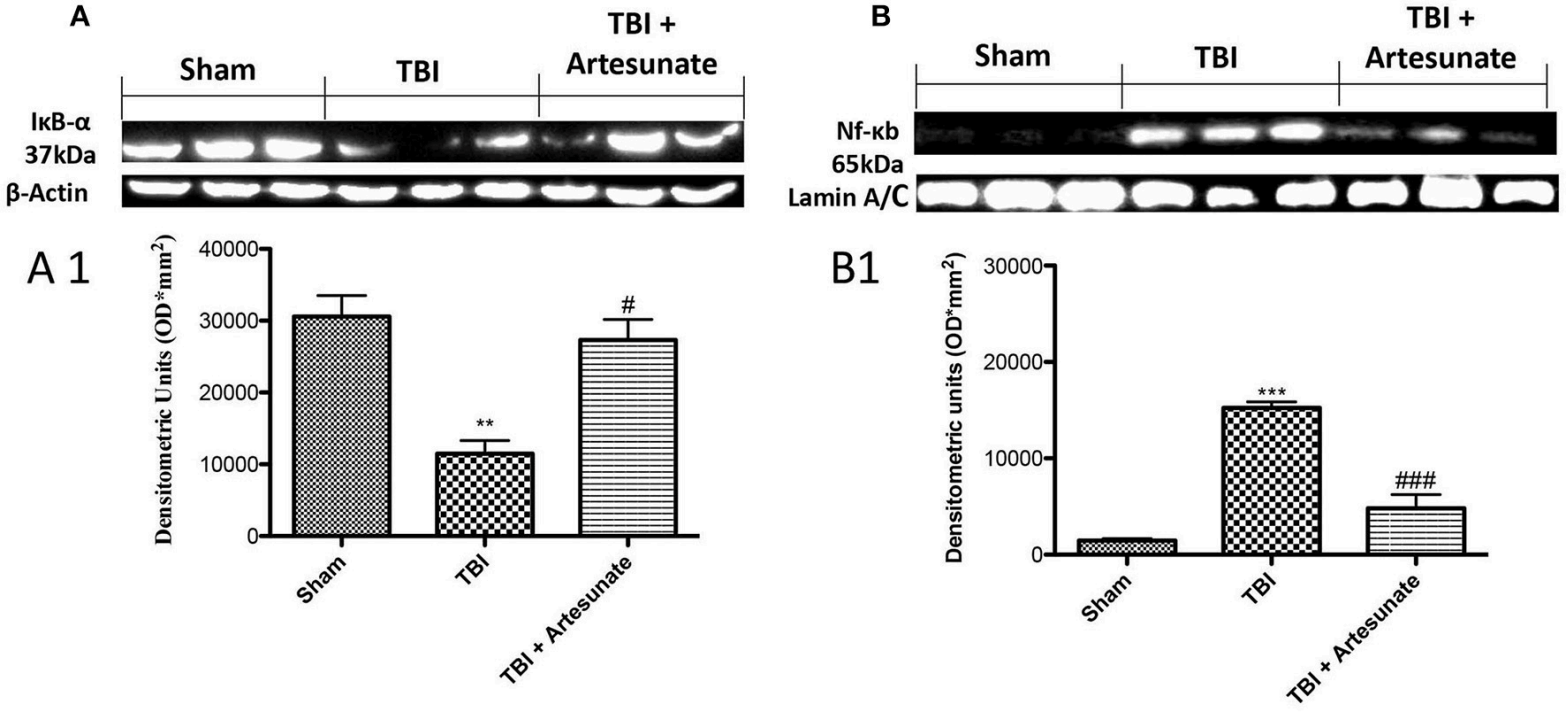
Protective effect of artesunate treatment on Iκb-α degradation and Nf-κb translocation. **(A)** Show the basal expression of IκB-α in brain homogenates from sham group, 24 h after TBI injury there is a significant reduction in IκB-α expression, while the treatment with artesunate was able to prevent reduction in IκB-α expression, see densitometric analysis **(A1)**. **(B)** Showed a significantly increase in Nf-κb expression 24 h after TBI injury, in vehicle group. When compared with vehicle group, treatment with artesunate 30 mg\Kg significantly reduce the Nf-κb expression see densitometric analysis **(B1)**. Data are expressed as Mean ± SEM from *N* = 10 Mice for each group. **P < 0.01 vs. respective sham; ***P < 0.001 vs. corresponding sham group. ^#^*P* < 0.05 vs. corresponding TBI. ^###^*P* < 0.001 vs. corresponding TBI. (*F*-value for IκB-α = 17.23. *df* = 2. *r*^2^ = 0.8733) (*F*-value for Nf-κb = 63.74. *df* = 2. *r*^2^ = 0.9550) (see the Supplementary Figures 1, 2 for the triplicate of western blots).

To test the anti-inflammatory proprieties of artesunate, we evaluated by western blot analysis the Iκ-α degradation and Nf-κb translocation, that play a significant role in inflammation. Figure 2A showed that after TBI injury in the vehicle group there is a significant degradation of Iκb-α compared to the sham group, the treatment with artesunate can significantly prevent the Iκb-α degradation compared to the vehicle group (Figures 2A,A1). Consequently, western blot analysis in the vehicle group showed a significant increase of Nf-κb translocation, compared to the sham group (Figures 2B,B1). On the contrary artesunate treatment significantly reduce the Nf-κb translocation as show in Figures 2B,B1.

### Protective effect of artesunate treatment on cytokines, and iNOS expression

**Figure 3:**
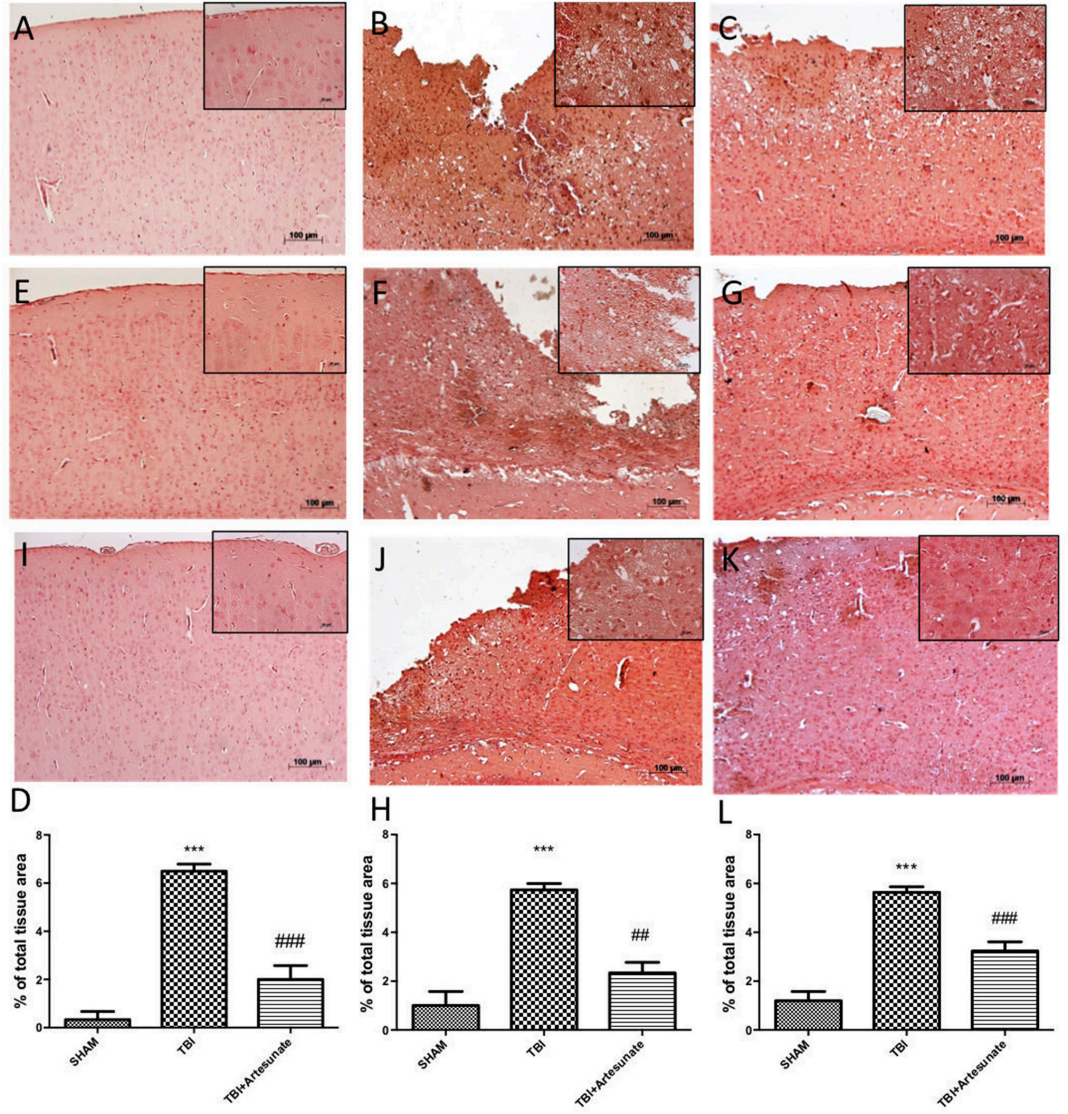
Protective effect of artesunate treatment on cytokines and iNOS expression. **(B,F)** Showed a significant increase of expression for IL-1β and TNF-α respectively, 24 h after TBI injury compared with sham group **(A,E)**. When compared with vehicle group, brain section from mice treated with artesunate show a significant reduction in IL-1β and TNF-α respectively **(C,G)**, see densitometric analysis **(D)** for IL-1β, and **(H)** for TNF-α. The immunohistochemical analysis for iNOS, show that 24 h after TBI injury in the vehicle group there is a significant increase in iNOS expression as show in **(J)** compared with sham group **(I)**. While as show in **(K)** treatment with artesunate significantly reduce the iNOS expression compared to vehicle group, see densitometric analysis **(L)**. Data are expressed as Mean ± SEM from *N* = 10 Mice for each group. ***P < 0.001 vs. corresponding sham group. ^##^*P* < 0.01 vs. respective TBI. ^###^*P* < 0.001 vs. corresponding TBI. (*F*-value for IL-1β = 57.84. *df* = 2. *r*^2^ = 0.9507) (*F*-value for TNF-α = 30.01. *df* = 2. *r*^2^ = 0.9091) (*F*-value for iNOS = 45.29. *df* = 2. *r*^2^ = 0.883).

Next, we evaluated the anti-neuroinflammatory effect of artesunate treatment post TBI, in cytokines expression. The immunohistochemistry analysis for Il-1β and TNF-α showed that, 24 h after TBI there is a significantly increase in Il-1β and TNF-α expression in brain tissue from vehicle group (Figures 3B,F respectively), when compared with brain from sham group (Figures 3A,E respectively), instead the treatment with artesunate significantly reduce the expression in Il-1β and TNF-α (Figures 3C,G respectively) when compared to vehicle as show in Figures 3D,H respectively. Moreover, we also assessed iNOS is expression in response to pro-inflammatory cytokines. Immunohistochemical analysis for iNOS expression shows that 24 h after TBI injury. In the vehicle group there is a significant increase in iNOS expression (Figure 3J see densitometric analysis Figure 3L) compared to sham group. (Figure 3I see densitometric analysis Figure 3L), showed that after treatment with artesunate 30 mg\Kg significantly reduce the iNOS expression when compared to the vehicle group, as show in Figure 3K (see densitometric analysis Figure 3L).

### Effect of artesunate on inflammasome components

**Figure 4:**
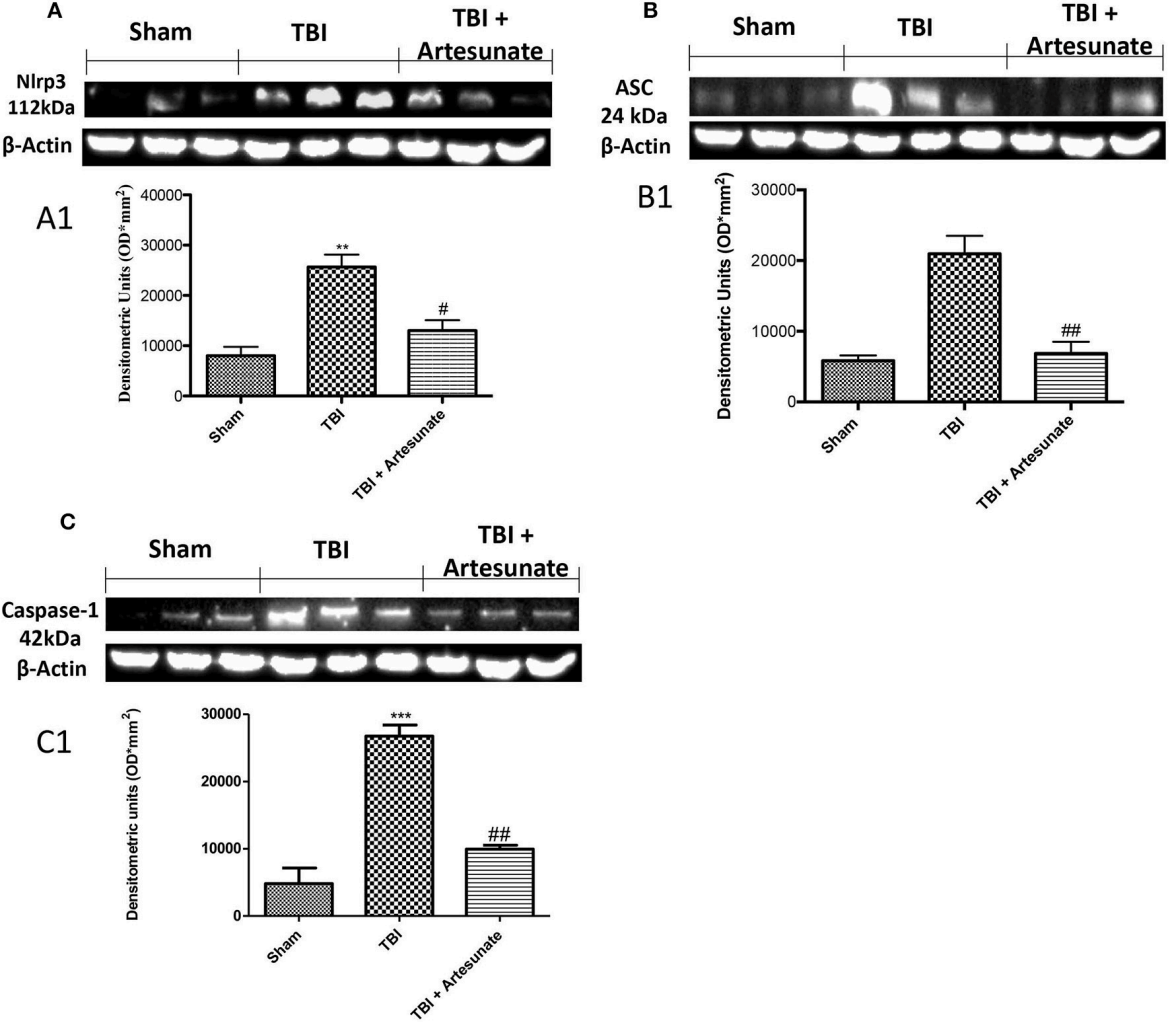
Effect of artesunate on inflammasome components. Western blot analysis show that 24 h after TBI injury in the TBI group there is a significantly increase in expressions of NLRP3 **(A)**, ASC **(B)**, and caspase-1 **(C)**. When compared with TBI group, treatment with artesunate significantly reduce the expression of inflammasome components as show in **(A)** for NLRP3 see densitometric analysis **(A1**,**B)** for ASC see densitometric analysis **(B1,C)** for Caspase-1 see densitometric analysis **(C1)**. Data are expressed as Mean ± SEM from *N* = 10 Mice for each group, **p < 0.01 vs. respective sham. ***P < 0.001 vs. corresponding sham group; ^#^*P* < 0.05 vs. corresponding TBI. ^##^*p* < 0.01 vs. corresponding TBI. (*F*-value for NLRP3 = 18.30. *df* = 2. *r*^2^ = 0.8591) (*F*-value for ASC = 23.14. *df* = 2. *r*^2^ = 0.8852) (*F*-value for Caspase-1 = 47.25. *df* = 2. *r*^2^ = 0.9403) (see the Supplementary Figures 3–5 for the triplicate of western blots).

NLRP3-inflammasome, which also includes adaptor proteins such as the apoptosis-associated speck-like protein containing a caspase-recruitment domain (ASC), and the serine protease caspase 1 (Casp1), play a key role in the inflammatory response. Western blot analysis of brain homogenates 24 h after TBI injury the TBI group shows a significant increase in NLRP3, ASC, and Caspase-1 expression compared to sham group. When compared with vehicle group treatment with artesunate at Dose of 30 mg\Kg significantly reduce the expressions of inflammasomes components expression NLRP3 (Figures 4A,A1), ASC (Figures 4B,B1) and Caspase-1 (Figures 4C,C1).

### Protective effect of artesunate treatment on apoptosis process

**Figure 5:**
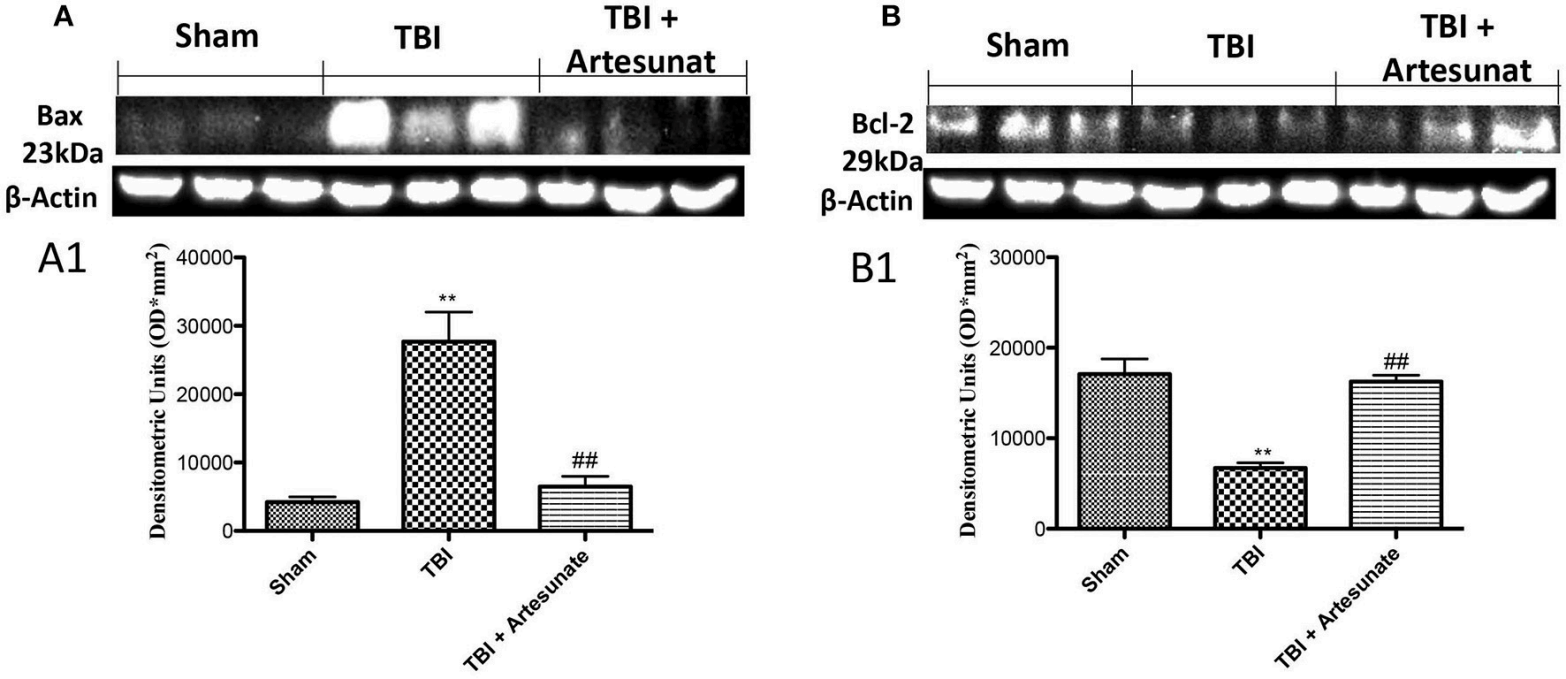
Protective effect of artesunate treatment on apoptosis process. **(A)** Western blot analysis for Bax showed that 24 h after TBI injury, brain from vehicle group showed a significant increase in Bax expression compared to sham group, instead brain from mice that have been treated with artesunate showed a significant reduction in Bax expression compared to vehicle group, see densitometric analysis **(A1)**. **(B)** Showed a basal expression of Bcl-2 in brain homogenates from sham mice, after TBI injury the vehicle group showed a significant reduction in Bcl-2 expressions. Treatment with artesunate was able to prevent the reduction in Bcl-2 expression and reports the levels similar to the sham group, see densitometric analysis **(B1)**. Data are expressed as Mean ± SEM from *N* = 10 Mice for each group **P < 0.01 vs. respective sham; ^##^*P* < 0.01 vs. respective TBI. (*F*-value for Bax = 22.93. *df* = 2. *r*^2^ = 0.8843) (*F*-value for Bcl-2 = 27.64. *df* = 2. *r*^2^ = 0.9021) (see the Supplementary Figures 6, 7 for the triplicate of western blots).

To test if the treatment with artesunate was able to prevent apoptosis process, brain homogenates, were processed for Western Blot analysis for pro-apoptotic factor Bax and anti-apoptotic factor Bcl-2 expression. Sham-operated mice showed a basal expression for Bcl-2 (Figures 5A,A1,B,B1). Instead the vehicle group showed a significantly reduction in Bcl-2 and significantly increases in Bax expression. Treatments with artesunate a dose of 30 mg\Kg were able to reduce the increase of Bax expression and to prevent the reduction in Bcl2 expression as show in Figures 5A,A1,B,B1.

### Protective effect of artesunate treatment on astrocytes and microglia activation and cytokine production

**Figure 6:**
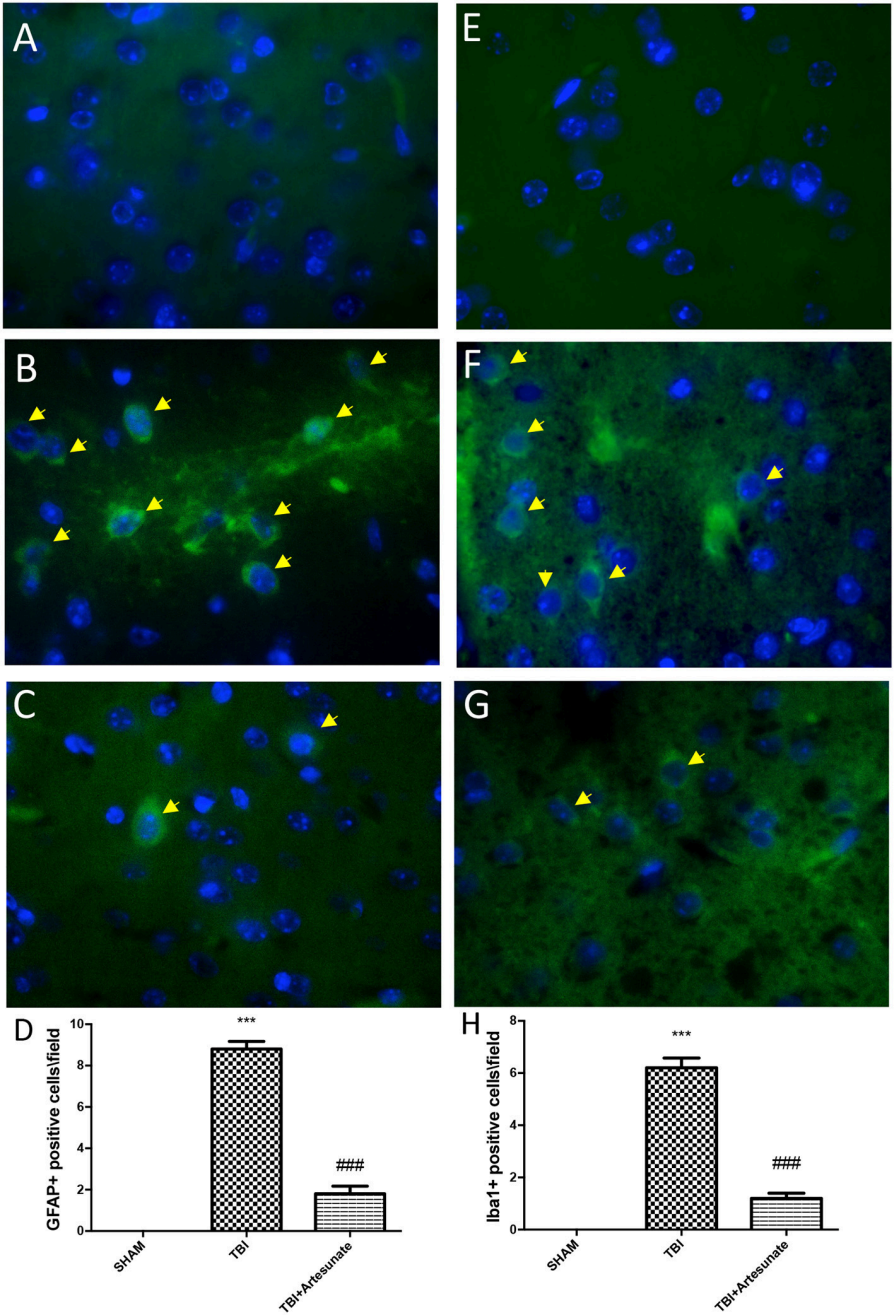
Protective effect of artesunate treatment on astrocytes and microglia activation. **(A,E)** Showed no immunoreactivity for GFAP and Iba-1 respectively in sham brain tissue. Twenty-four hours after TBI injury in brain tissue there is a significant increase in positive cells for GFAP and Iba-1 as show in **(B,F)** respectively, see yellow arrows. When compared with TBI group treatment with artesunate significantly reduce the number of positive cell for GFAP and Iba-1as show in figure **(C,G)** respectively, see yellow arrows. **(D)** GFAP densities measured, panels. **(H)** Iba-1 densities measured. Data are expressed as Mean ± SEM from *N* = 10 Mice for each group, ***P < 0.001 vs. corresponding sham group. ^###^*P* < 0.001 vs. corresponding TBI. (*F*-value for GFAP = 231.6. *df* = 2. *r*^2^ = 0.9747) (*F*-value for IBA-1 = 180.2. *df* = 2. *r*^2^ = 0.9678).

Astrocytes and microglia activation play a critical role in neuroinflammation. When compared to the sham group (Figure 6A, for GFAP and Figure 6E for Iba-1, see yellow arrows), immunofluorescence evaluation of Glial fibrillary acidic protein (GFAP) and ionized calcium binding adaptor molecule 1 (Iba1) revealed a significant increasing in GFAP and Iba-1 positive cell as show in Figures 6B,F respectively. Treatment with artesunate at dose of 30 mg\Kg post TBI injury significantly reduce the number of positive cells for GFAP (Figure 6D) and Iba-1 (Figures 6C,G,H) respectively, compared to the vehicle group.

**Figure 7:**
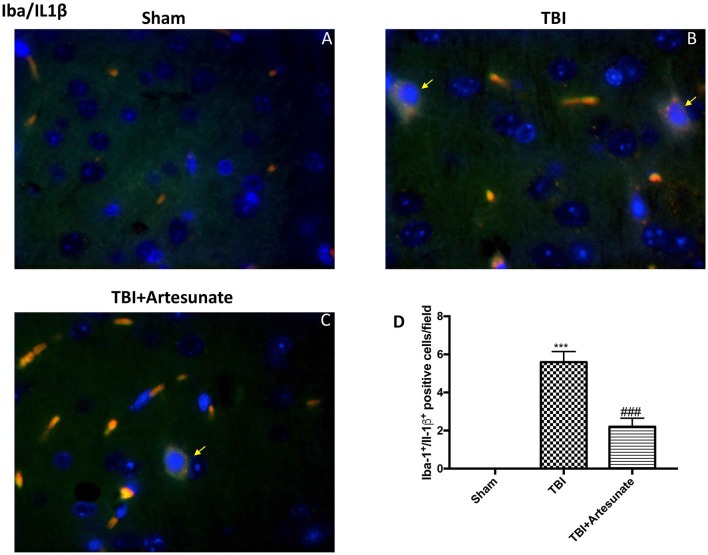
Protective effect of artesunate treatment on microglial cell activation and cytokine production. **(A)** Showed no immunoreactivity for Iba-1+/IL-1β+ in sham group. **(B)** Showed a significant increase in IBA-1+ /IL-1β+ co-localization. **(C)** Treatment with artesunate significantly reduced the TBI-induced expression of IBA-1+/IL-1β+. **(D)** Represent densitometric analysis. The yellow arrow indicates the co-localization between IBA-1+/IL-1β+. Data are expressed as Mean ± SEM from *N* = 10 Mice for each group, ***P < 0.001 vs. corresponding sham group. ^###^*P* < 0.001 vs. corresponding TBI. (*F*-value = 238.8. *df* = 2. *r*^2^ = 0.9755).

Moreover, we investigated by double co-localization the expression of IL-1 β with the specific marker for microglia. We observed that IBA-1+/IL-1β+ co-localization was very low in the sham group (Figure 7A; densitometric analysis, Figure 7D), but significantly elevated following TBI injury (Figure 7B; densitometric analysis, Figure 7D). Artesunate treatment (30 mg/kg) significantly reduced the TBI-induced expression of IBA-1+/IL-1β+ (Figure 7C; densitometric analysis, Figure 7D).

### Protective effect of artesunate treatment on neutrophic factors

**Figure 8:**
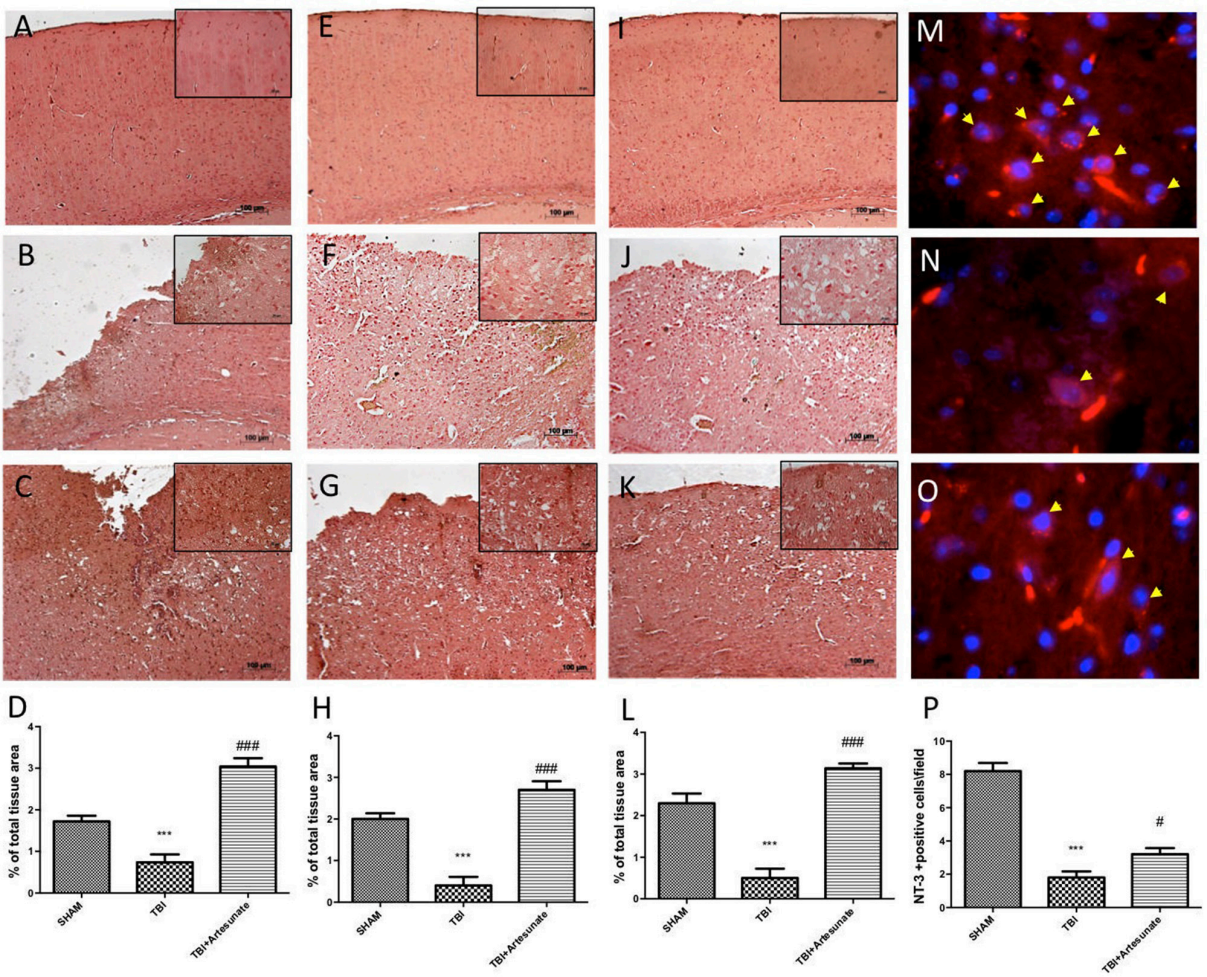
Protective effect of artesunate treatment on neutrophic factors. **(A)** Show the immunohistochemical expression of VEGF in brain section from sham group, **(B)** show a significant reduction in VEGF expression 24 h after TBI injury in brain section from vehicle group, instead **(C)** show that treatment with artesunate significantly increase the VEGF expression compared with the vehicle group, **(D)** VEGF density. **(E,I)** Show basal expression for BDNF and GDNF respectively in brain section from sham mice. 24 h after TBI there is a significantly reduction in BDNF and GDNF expression as show in **(F,J)** respectively, instead **(G,K)** show that treatment with artesunate is able to significantly increase the expression of BDNF and GDNF respectively **(H,L)** BDNF and GDNF density. **(M)** Show the basal NT-3 immunoreactivity (red signal) in brain from sham mice. **(N)** Brain tissue from TBI group showed a significant reduction in NT-3 levels. **(O)** When compared with TBI group the brain section from mice that have been treated with artesunate showed a significant increase in NT-3 levels. **(P)**, NT-3 density. Data are expressed as Mean ± SEM from *N* = 10 Mice for each group, ***P < 0.001 vs. corresponding sham group. ^#^*P* < 0.05 vs. corresponding TBI. ^###^*P* < 0.001 vs. corresponding TBI. (*F*-value for VEGF = 41.8. *df* = 2. *r*^2^ = 0.8745) (*F*-value for BDNF = 38.97m. *df* = 2. *r*^2^ = 0.8666) (*F*-value for GDNF = 46.41. *df* = 2. *r*^2^ = 0.8855) (*F*-value for NT-3 = 65.31. df = 2. *r*^2^ = 0.9159).

Neurotrophic factors play a key role in neuronal survival and repair processes following damage to the brain. First, by immunohistochemical analysis we evaluated the effects of artesunate on the expression of VEGF, which plays a key role in promoting the repair of damaged tissues. 24 h after the TBI, the vehicle group shows reduced levels of VEGF compared to sham as show in Figures 8A,B respectively, instead following the treatment with artesunate (Figure 8C) there is a significant increase in the expression of VEGF compared to the vehicle group as show in Figure 8D. Figures 8E,I showed the basal expression of Brain-derived neurotrophic factor (BDNF) and Glial cell-derived neurotrophic factor (GDNF) respectively, brain section from sham animal. 24 h after TBI injury we found a significantly reduction in BDNF and GDNF expression in brain from vehicle group mice as show in Figures 8F,J respectively. While treatment with artesunate at dose of 30 mg\Kg was able to significantly increase the expression of BDNF and GDNF (Figures 8G,K respectively) after TBI injury as show in Figures 8H,L. Subsequently, by immunofluorescence analysis we evaluated the expression of neurotrophic factor Neurotrophin-3 (NT-3). The Figure 8M shows the basal expressions of NT-3 in the brain section from sham mice. After the TBI injury, there is a significant reduction in NT-3 expression in the brain section from vehicle group as show in Figure 8N. instead the treatment with artesunate significantly prevent the reduction in NT-3 expression induced by TBI, Figures 8O,P.

**Figure 9:**
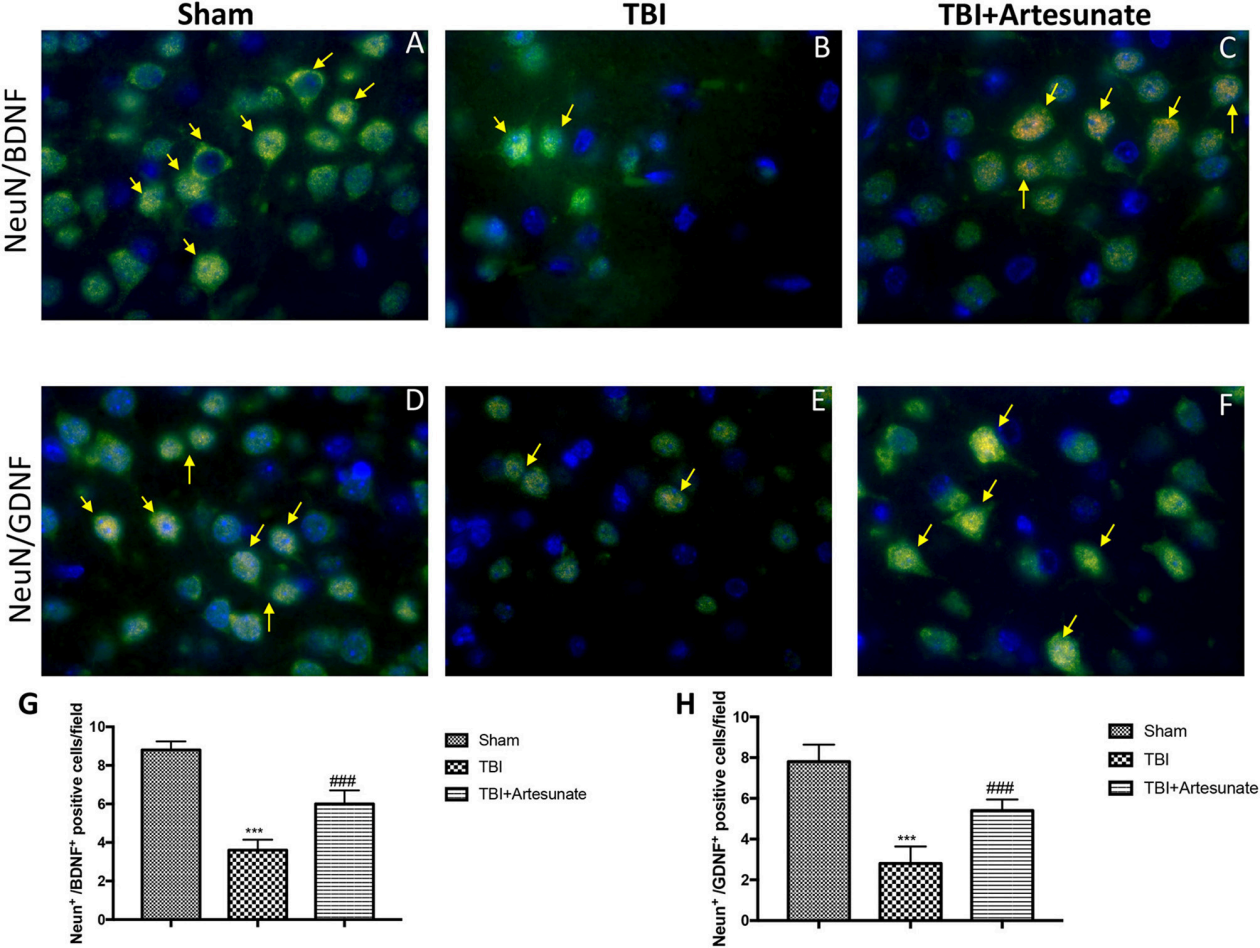
Protective effect of artesunate treatment on co-localization between BDNF and GDNF with Neu-N. **(A,D)** Showed the co-localization between BDNF+/Neu-N+ and GDNF+/Neu-N+ respectively in sham brain tissue. Twenty-four hours after TBI injury in brain tissue there is a significant decrease in positive cells for BDNF+/Neu-N+ and GDNF+/Neu-N+ as show in **(B,E)** respectively. Treatment with artesunate significantly increased the release of BDNF and GDNF as show in figure **(C,F)** respectively. **(G,H)** Represent, respectively, densitometric analysis. The yellow arrow indicates the co-localization between BDNF and Neu-N and GDNF and Neu-N. Data are expressed as Mean ± SEM from *N* = 10 Mice for each group, ***P < 0.001 vs. corresponding sham group. ^###^*P* < 0.001 vs. corresponding TBI. (*F*-value for BDNF+/Neu-N+ = 101.6. *df* = 2. *r*^2^ = 0.9442) (*F*-value for GDNF+/Neu-N+ = 55.18. *df* = 2. *r*^2^ = 0.9019).

Moreover, we investigated by double co-localization the expression of BDNF and GDNF with Neu-N, a marker for neuronal nuclei. BDNF and GDNF expressions were decreased in animals after TBI injury (Figures 9B,E respectively, densitometric analysis, Figures 9G,H) compared to sham group (Figures 9A,D respectively, densitometric analysis Figures 9G,H), while treatment with artesunate significantly increased the release of BDNF and GDNF (Figures 9C,F respectively, densitometric analysis Figures 9G,H). The yellow arrow indicates the co-localization between BDNF and Neu-N and GDNF and Neu-N.

## Discussion

Traumatic brain injury is a leading cause of mortality and is a major public health issue. Currently, most of the drugs available for the treatment of TBI have as a target, a single aspect of the lesion. It is widely recognized that the etiopathology events in the TBI comprise a series of heterogeneous events, which include cellular and molecular mechanisms both in the acute and non-acute phases, thus making it difficult to achieve a single pharmacological treatment. In fact, effective treatment for the TBI should simultaneously attenuate several injury factors. Thus, attenuate the inflammatory process at the base of the pathological process, and prevent the neurodegeneration and cognitive decline that the TBI involves, is a fundamental step in the treatment of the TBI (15). Agents with more than one protective effect are attractive as potential therapeutic drugs for neurological disorders. Therefore, in this sense Artesunate appears to be a good candidate for the treatment of central nervous system diseases (11). Artesunate is a derivative obtained from a Chinese plant Artemisia annua. Currently it is the drug used for the treatment of resistant malaria and for brain malaria, also saw its good safety profile without side effects. Until now, controlled cortical impact (CCI) represents the most frequently used mechanical model to induce TBI, given its accuracy, easy of control, and, most importantly, its ability to produce brain injuries similar to those seen in humans (30). in this study we evaluated the treatment with artesunate at a dose of 30 mg\kg in the modulation of the inflammatory process, as in the activation of Nf-kb, proinflammatory cytokines IL-1β, TNF-α, and iNOS expression. We also evaluated the protective effects of artesunate in the activation of the NLRP3 inflammasome complex, which plays a key role in the inflammatory process typical of the second phase of the TBI. We also evaluated the protective effects of artesunate in promoting reparative processes (VEGF), and in preventing neurodegeneration through the modulation of neurotrophic factors such as BDNF GDNF and NT-3. Histological analysis shows how the artesunate treatment had a significant protective effect in this experimental model of TBI. In fact, we have seen how the group treated with artesunate showed a reduced lesion area, and a minor morphological modification respect to the vehicle group, following the TBI. Then first, we evaluated the treatment with artesunate in inhibiting the inflammatory process subsequently at TBI. In fact, neuroinflammation is a secondary process that contributes to tissue damage and neurological disorders that the TBI involves. Some research has shown that the presence of neuroinflammation after brain trauma can play a key role in the loss of neurological function (31, 32). The brain trauma causes an alteration of the normal function of the blood brain barrier, thus allowing access to the lesion site, circulating macrophage neutrophil and lymphocytes, therefore the accumulation of these cells releases inflammatory mediators that activate the glial and inflammatory cells, thus supporting the inflammatory process (33, 34). The transcription factor nuclear factor kappa B (NF-κB) is a master regulator of inflammation. It also mediates a variety of other cellular processes including cell survival and apoptosis, the activity includes those engaged by neurotrophic factors, neurotransmitters, electrical activity, cytokines, and oxidative stress. Emerging findings support a pivotal role for NF-κB as a mediator of transcription-dependent enduring changes in the structure and function of neuronal circuits (35). NF-κB activity is tightly regulated. It is generally bound by the principal inhibitory protein, Iκ-bα, and is sequestered in the cytoplasm. NF-κB can be activated by cytokines (like TNF-α) or other stimuli including trauma. This requires the degradation of Iκ-Bα, thereby freeing NF-κB to translocate to the nucleus. Moreover, NF-κB is important for the regulation of other enzymes such as those involved in the worsening of oxidative stress damage during the TBI. Here in this model of TBI we found that 24 h after TBI injury there was a significant degeneration of Iκ-bα and a consequent increase in NF-κB translocation. Instead the treatment with artesunate was able to prevent the degradation of Iκ-bα and the translocation of NF-κB. Moreover, the up-regulation of proinflammatory cytokines like IL-1β and TNF-α, oxidative stress, immune cells proteases and toxic metabolites can cause additional tissue damage that subsequently, provokes neuronal cell death and gradual axonal loss over time (36, 37). Our results show that 24 h after the TBI, in the brains of the mice from the vehicle group there was a significant increase in the expression of IL1-β and TNF-α, and that treatment with artesunate significantly reduced the expression of these proinflammatory cytokines after TBI. The iNOS is responsible for the production of reactive nitrogen species, which cause various types of cell damage including DNA damage (38, 39). Therefore, the increase in the expression of iNOS can weaken the oxidative stress damage during the secondary events to the TBI. in fact an increased expression of iNOS has been found in the areas associated with inflammatory processes after TBI injury (40), where it plays a role in secondary injury from TBI. Therefore, inhibition of iNOS expression has a protective role against TBI damage (41). Our results show an increase in iNOS expression 24 h after the TBI, while the treatment with artesunate at a dose of 30 mg\Kg was able to prevent the increase of iNOS expression. Recently it has been highlighted as nucleotide-binding domain, leucine-rich repeat, pyrin domain containing 3 (NLRP3), a key component of the NLRP3-inflammasome, mediates the inflammatory response, and therefore plays a key role in the secondary injury of the TBI (42, 43). The inflammasome is a multiprotein complex involved in innate immunity and in a host inflammatory signaling. NLRP3-inflammasome, which also include adaptor proteins such as the apoptosis-associated speck-like protein containing a caspase-recruitment domain (ASC), and the serine protease caspase 1 (Casp1) (44). the activation of NLRP3 thus leads to the maturation of Capase-1 which is subsequently responsible for the release of the proinflammatory cytokines IL-1β and IL-18. These cytokines play a key role in mediating the immune and inflammatory response, therefore NLRP3 activation can modulates neuroinflammation and neurodegenerative processes, as in the TBI (45). In fact, recently it has been seen that inhibition of NLRP3 pathway can help to prevent the pathological events resulting from the TBI (19, 46). In this study, we have seen that, in the vehicle group there is a significant activation of the inflammasome complex, as is evident from the increase in the expression of its components NLRP3 ASC and caspase −1. Instead the treatment with artesunate was able to significantly reduce the increase in the expression of inflammasomes components, induced by the TBI. Moreover, the major focus of TBI research should be the protection of neurons from apoptotic cell death by reducing the secondary injury of inflammation and oxidative stress (47). The continuous research for new therapeutic strategies for the treatment of TBI, have shown that it is essential to have as its objective the inhibition of the inflammatory process and the reduction of oxidative stress (48). In fact, these conditions are the most responsible for worsening of post-TBI events such as tissue damage and consequent apoptosis, responsible for neuronal death and alterations in neuronal function (49). The apoptotic process, is an important type of programmed cell death activated in the post TBI (50, 51). Therefore, an inhibition of this process is a fundamental step in the treatment of TBI and in preventing the decline of neurological functions. In this study we evaluated whether treatment with artesunate was able to mitigate the induced TBI apoptotic process, by evaluating the expression of two of the factors involved in the regulation of the apoptotic process BAX and Bcl-2. Bax with pro-apoptotic activity is responsible for the development of neuronal death (52), while Bcl-2 has an anti-apoptotic action and therefore protective for neuronal cells (53). Our results show that after 24 h from TBI there was an increase in the expression of bax and a significant decrease of bcl-2 thus highlighting an imbalance toward the apoptotic pathway. Instead the treatment with artesunate has significantly reduced the levels of bax and reported the expression of bcl-2 like the sham group. A typical sign of damage to the central nervous system is the increase in reactive astrocytes as indicated by an increase in GFAP expression (54), moreover a significant increase in GFAP may be due to pathological processes in the central nervous system (55). It has been seen how the neuroinflammation in the TBI sees the involvement of microglia, showed by the increase in the expression of Iba-1 (56). Our results show a significant increase in the expression of GFAP and Iba-1 following TBI, while the treatment with artesunate was able to reduce the increase in GFAP and Iba-1, induced by TBI. The ability of damaged neurons to recover from injury, depends on the expression of genes related to survival and growth. These signals include neurotrophic factors that play a key role in neuronal survival and growth. Therefore, in this experimental model of TBI we examined whether the potential protective effects of artesunate, are also due to its ability to modulate the expression of neurotrophic factors. First of these we evaluated the expression of VEGF, in fact VEGF an angiogenic factor, is also responsible for the neurovascularization which is a fundamental step in the repair of brain tissue and for nerve regeneration (57). Our results show that, 24 h after the TBI, the group treated with artesunate showed a significant increase in the expression of VEGF compared to the vehicle group, thus indicating that artesunate is grateful to improve the reparative processes following TBI. Neurotropic factor such as BDNF, NT-3 and GDNF, are known to play a key role in neuronal development and regeneration, in fact it has been seen that these factors promote neuronal survival following a mechanic damage as in the TBI (58, 59). Our results show that treatment with artesunate, in addition to decreasing the inflammatory process typical of the second phase of TBI, can promote neuronal survival and regeneration, a fundamental event in preventing neurodegeneration, and therefore the decline of neuronal functions that the TBI involves. In fact, our results show that the treatment with artesunate is able to significantly increase the expression of the BDNF, GDNF, and NT-3 neurotrophins respect to the vehicle group, following the TBI. Therefore, our results taken together show that the treatment with artesunate has a protective effect against the secondary events after the TBI. In particular we have seen that artesunate is able to inhibit the inflammatory response and neuronal death, moreover the protective effects of artesunate are expressed through the promotion of neurotrophic factors, important for neuronal survival and in the reparative processes of the central nervous system.

## Data availability statement

All datasets (Generated\Analyzed) for this study are included in the manuscript and the Supplementary Files.

## Author contributions

EG: drafted the work, performed the experiments and approved the version to be published; RD, MC, and RF: performed the experiments and approved the version to be published; RS, RC, and DI: analyzed data, revised the manuscript and approved the version to be published; RDP and SC: revised it critically for important intellectual content and approved the version to be published.

### Conflict of interest statement

The authors declare that the research was conducted in the absence of any commercial or financial relationships that could be construed as a potential conflict of interest. The handling Editor declared a shared affiliation, though no other collaboration, with the authors.
