# Supplementary figures and images for: Neuroprotective Effect of Artesunate in Experimental Model of Traumatic Brain Injury
Source: Front Neurol. 2018 Jul 31;9:590. doi: 10.3389/fneur.2018.00590 (PMC6079305; doi:10.3389/fneur.2018.00590)

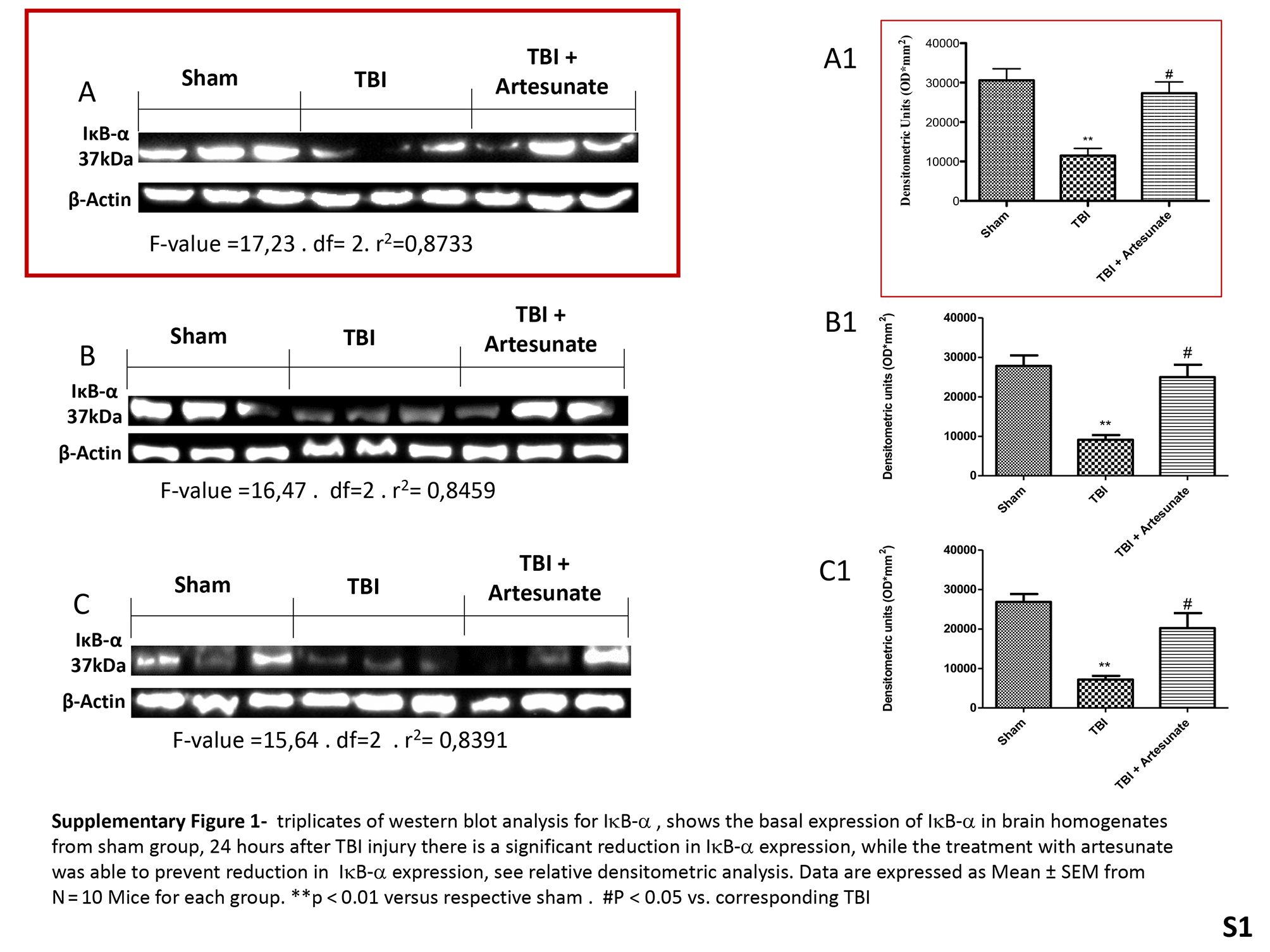

Supplement: Supplementary file 1 [file Image_1.tif]

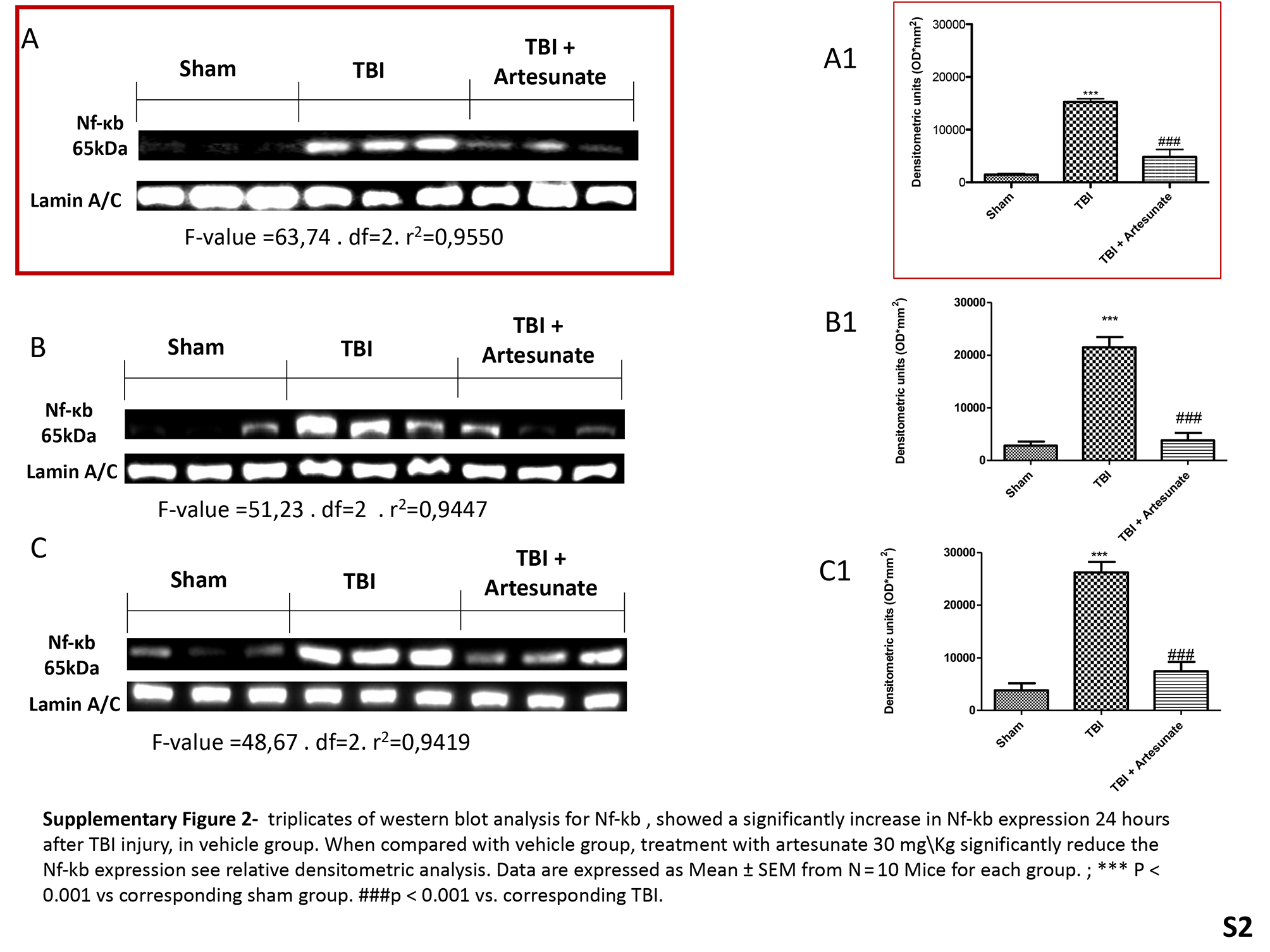

Supplement: Supplementary file 2 [file Image_2.tif]

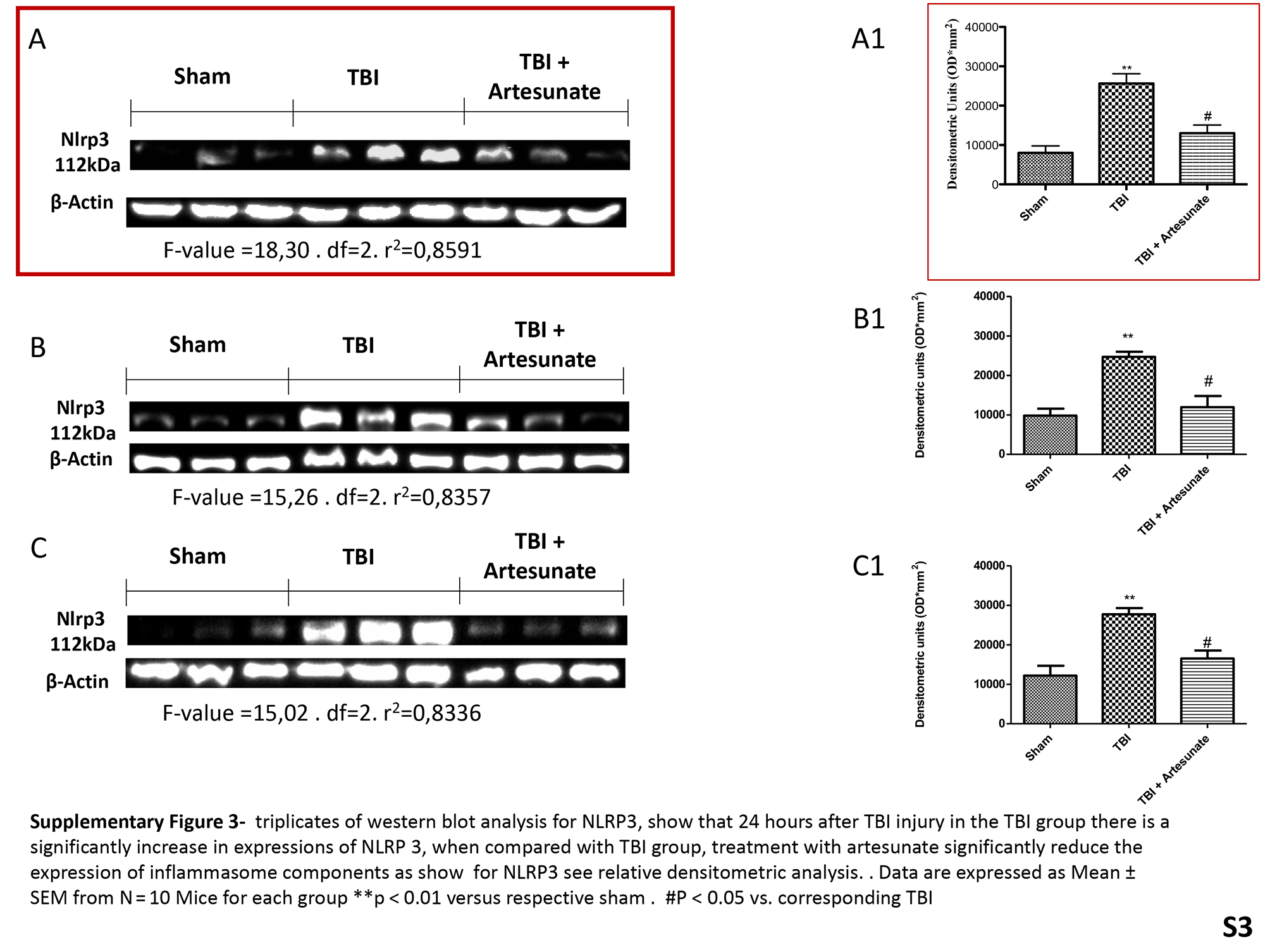

Supplement: Supplementary file 3 [file Image_3.tif]

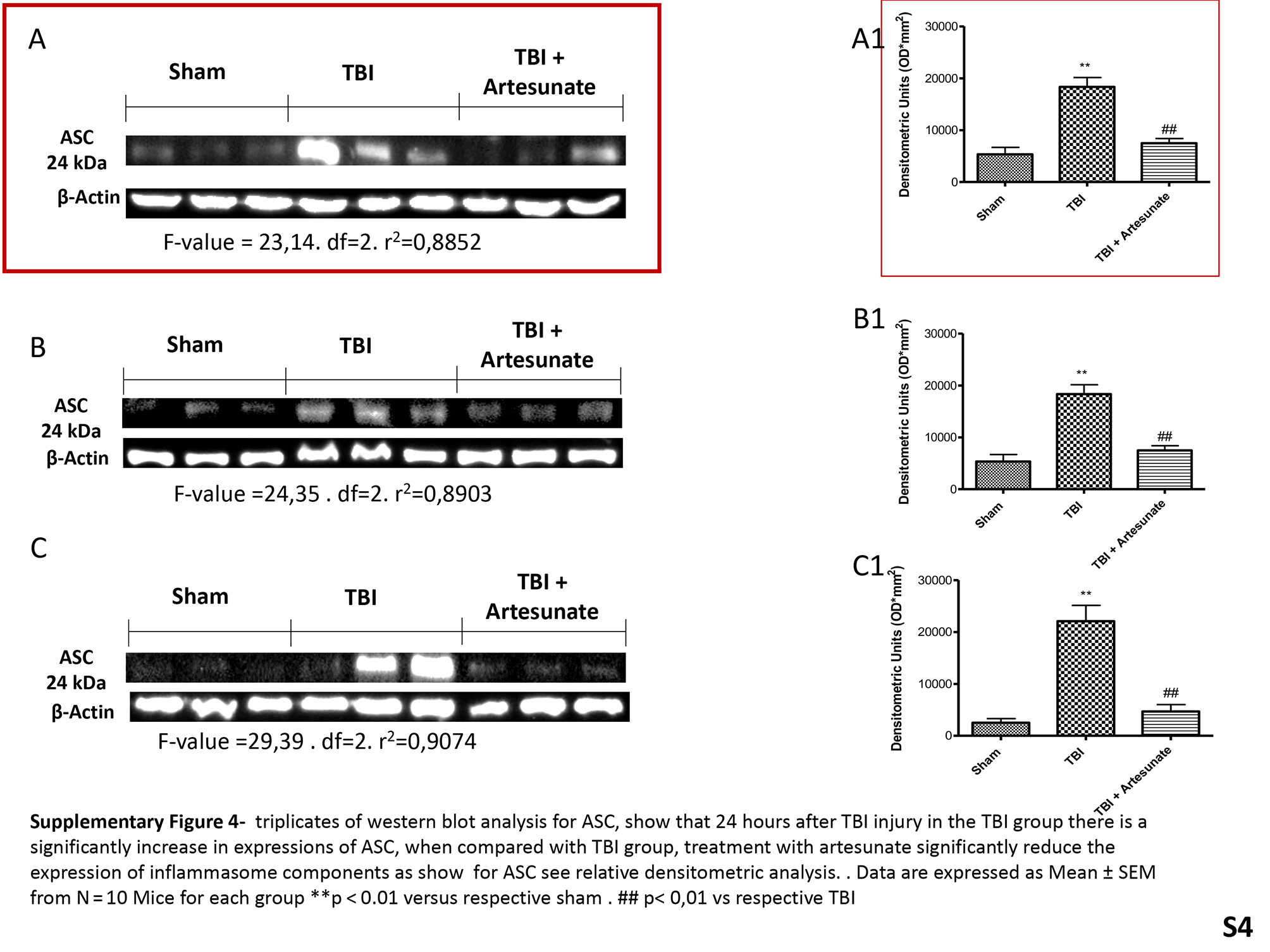

Supplement: Supplementary file 4 [file Image_4.tif]

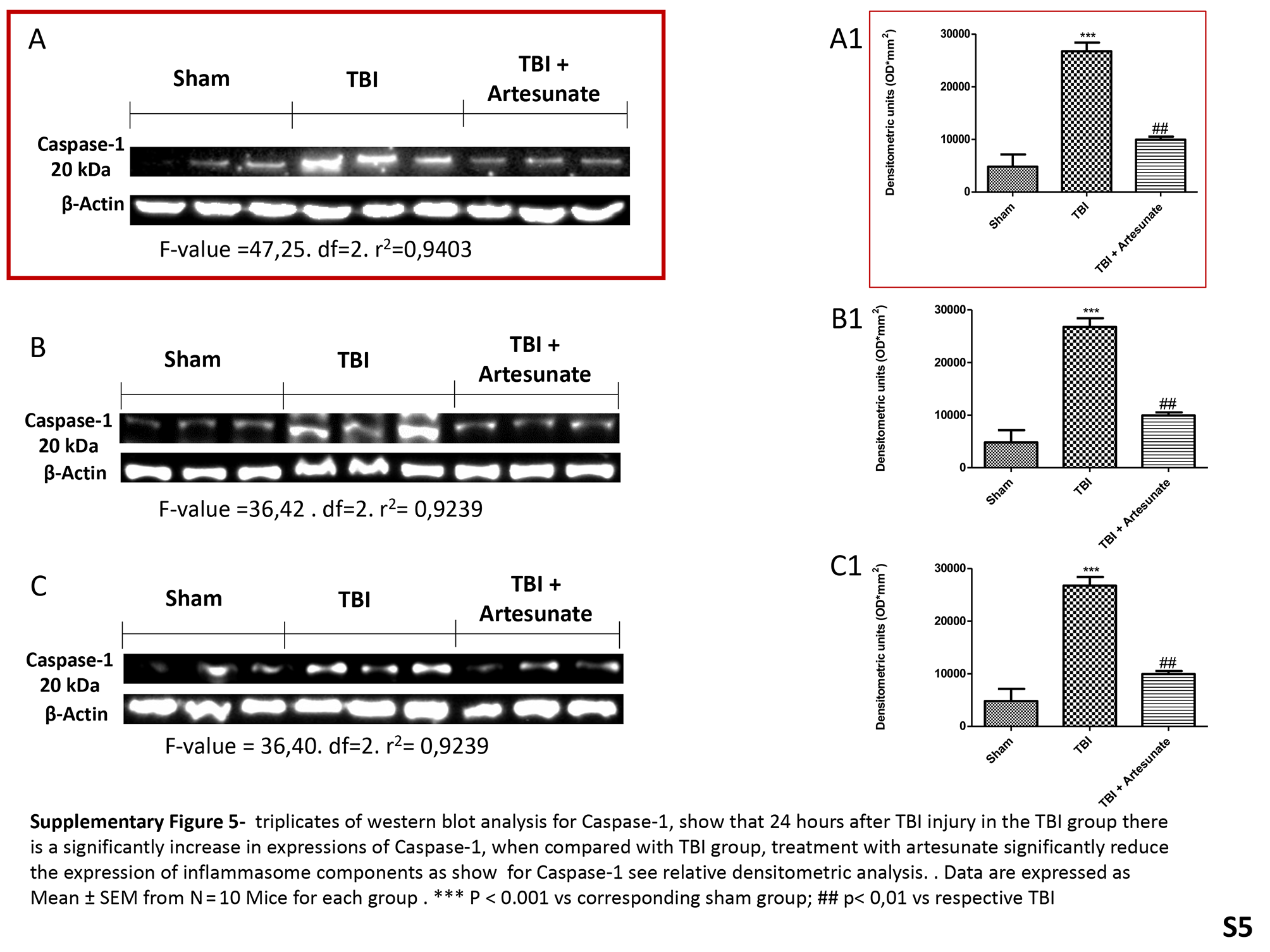

Supplement: Supplementary file 5 [file Image_5.tif]

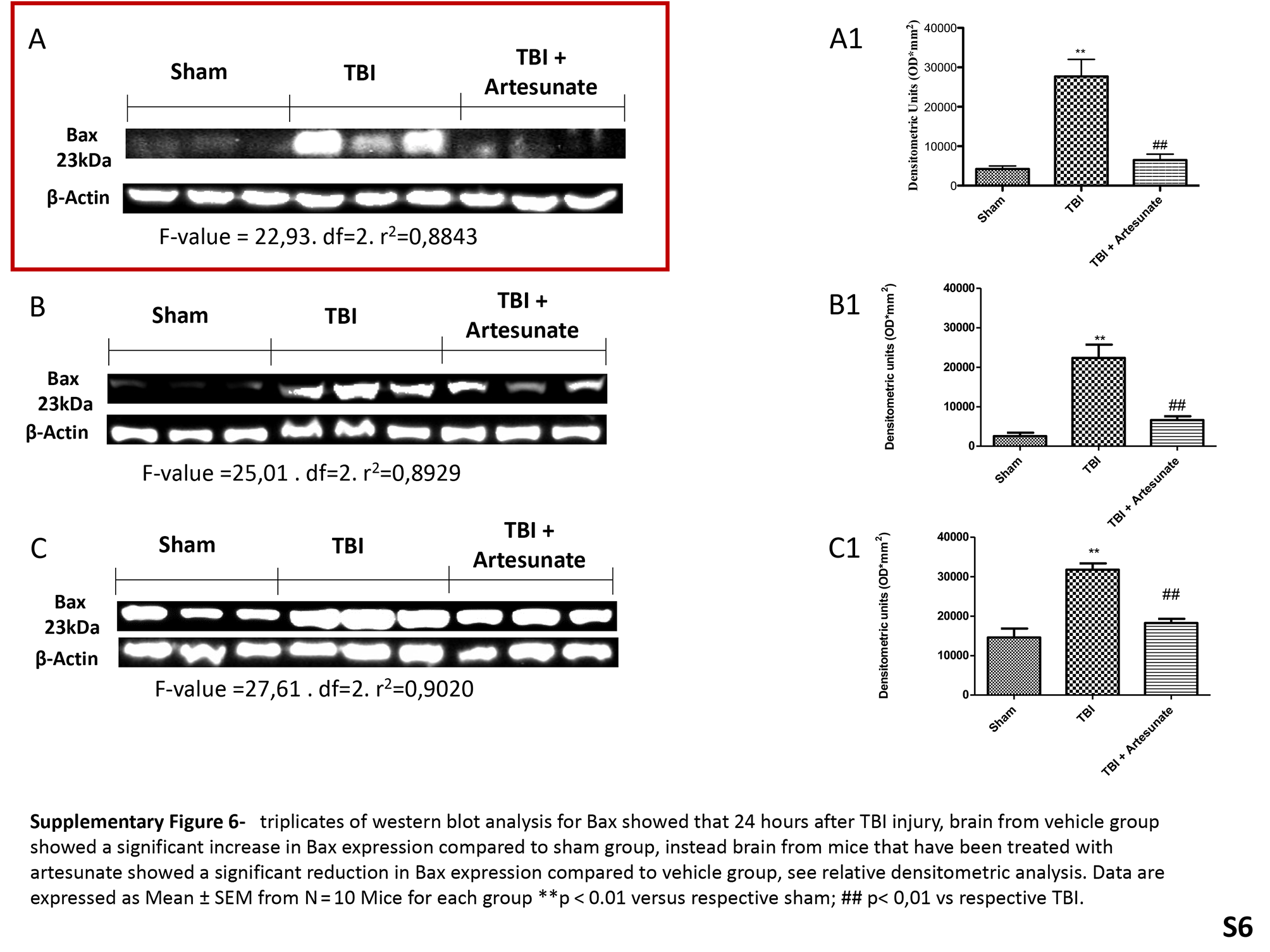

Supplement: Supplementary file 6 [file Image_6.tif]

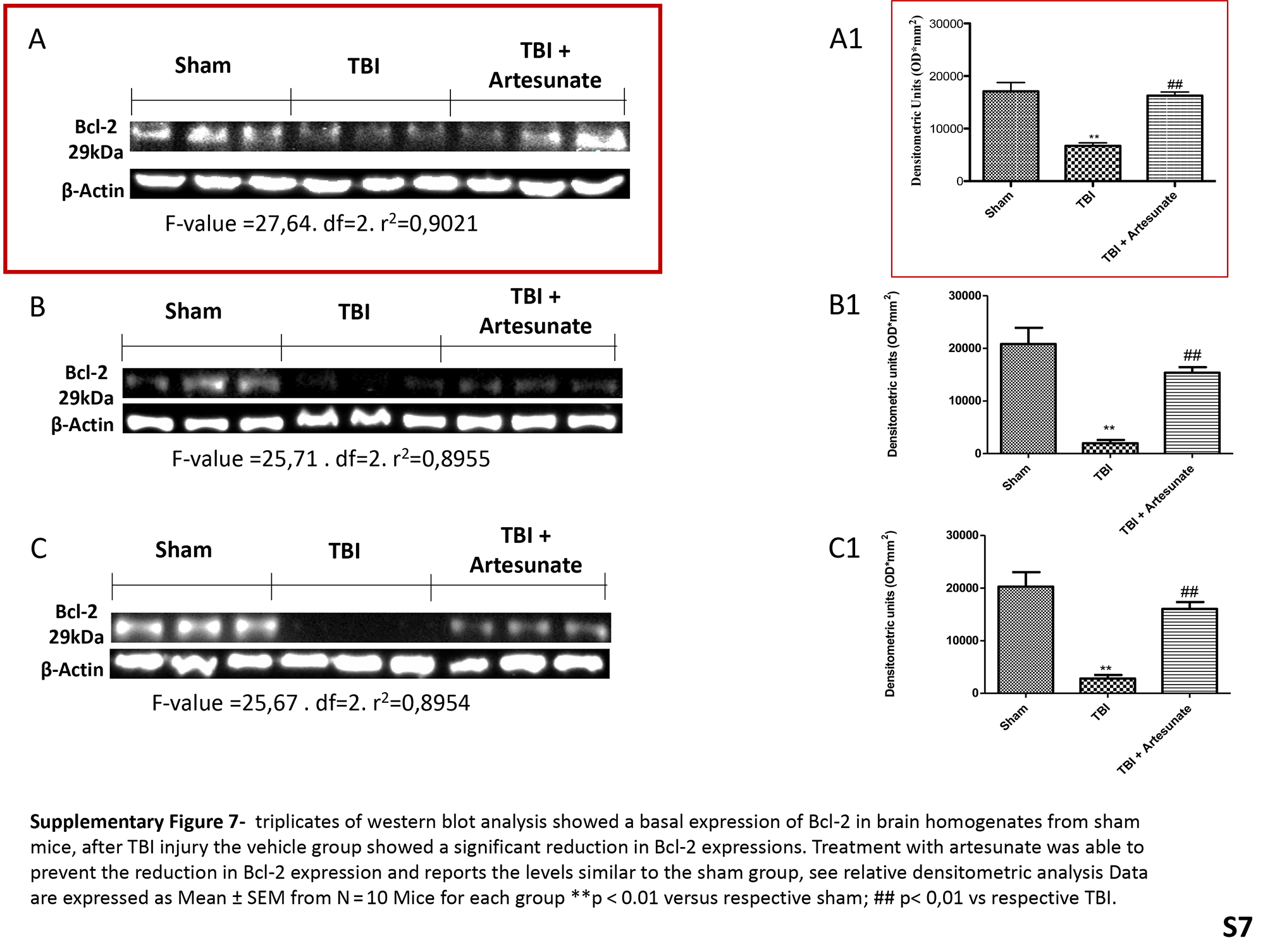

Supplement: Supplementary file 7 [file Image_7.tif]
